# Daily Text-Message Retrieval Practice for Pediatric Residents

**DOI:** 10.7759/cureus.95568

**Published:** 2025-10-28

**Authors:** Adin Nelson, Molly Broder, Alan Schwartz, Charlene Thomas, Erika Abramson

**Affiliations:** 1 Pediatrics, Weill Cornell Medicine, New York, USA; 2 Pediatrics, BronxCare Health System, New York, USA; 3 Medical Education, University of Illinois Chicago, Chicago, USA; 4 Biostatistics, Population Health Sciences, Weill Cornell Medicine, New York, USA

**Keywords:** education, exam preparation, graduate medical education, learning, residency, retrieval practice

## Abstract

Objective

Residents have limited time and much to learn. This creates an inescapable time pressure, which encourages reliance on efficient and effective learning strategies. Retrieval practice - study strategies such as flashcards and practice questions that demand active recall of desired information - is an increasingly common learning strategy for medical trainees. Growing evidence has linked retrieval practice to improved learning outcomes, but it has primarily come from small, observational studies, and their methods have varied widely. The variability in the existing literature makes it difficult to synthesize the evidence and identify best practices. We designed a national, prospective study to test the effects of daily retrieval practice for pediatric residents.

Methods

We recruited a national sample of pediatric residents in the United States. We sent participating residents mass text messages every morning for a year, directing them to answer one exam-style, multiple-choice, retrieval practice question. We then compared residents’ scores on national standardized exams at the beginning and end of the study period, and we compared scores between participating residents and a control group of residents not enrolled in the study.

Results

A total of 293 residents enrolled in the study. At baseline, their exam scores were no different from control residents’ scores. The daily mass text messages did not successfully induce residents to answer practice questions; most participants answered very few, and many answered none at all. There was no significant difference between participating residents’ and control residents’ exam scores, and there was no significant correlation between the number of questions participants answered and the change in their exam scores.

Conclusions

Previous observational studies have shown that the more exam-style practice questions residents answer, the better they score on subsequent exams. Small studies have also found that text message reminders encouraged residents to answer more practice questions and boosted their exam performance. In this large, national, prospective study, however, we found that daily text messages were not effective, and we were therefore unable to test how answering practice questions might have impacted residents’ annual standardized exam scores. Previous studies of text message retrieval practice may have missed or undervalued key elements of the text messaging programs that did not scale effectively to a national intervention. Future studies should explore effective strategies for increasing residents’ engagement with practice questions and other forms of retrieval practice.

## Introduction

Residents have limited time to acquire the medical knowledge and clinical skills they need to become independent clinicians and pass high-stakes licensing and certification examinations. That inherent time pressure pushes residents and residency educators to prioritize effective and efficient learning strategies. Retrieval practice is the technique of studying with activities such as quizzes, flashcards, and practice questions that require learners to actively recall desired information from memory rather than passively hearing or reading it [1], and it is one example of an evidence-based, effective and efficient, learning strategy [2] that has become increasingly common among medical students and residents [3,4]. Since the COVID-19 pandemic, retrieval practice using digital flashcards and exam-style multiple-choice practice questions has become even more common among a new generation of trainees who are more accustomed to and more reliant on self-directed asynchronous learning than previous trainees have been [5-7].

There is a growing body of evidence on the benefits of retrieval practice in Health Professions Education (HPE), but much of it comes from observational research on trainees’ independent studying. For example, studies have shown that the more exam-style multiple choice practice questions students [3] and residents [8] answered, the higher they scored on a subsequent standardized test, and that simply giving residents access to a bank of practice questions improved their exam scores [9].

There have been prospective studies of retrieval practice in HPE, but their methods have varied widely. On a small scale, for example, studies have shown that adding brief quizzes to the end of lectures enhanced residents’ long-term retention [10] and that a combination of weekly group practice question sessions and daily individual practice questions distributed by text message improved residents’ exam scores [11]. In larger studies, introducing biweekly online quizzes [12] and daily practice questions sent to residents’ mobile phones [13] boosted standardized test scores in whole residency programs, but those studies were not designed to test effects at the level of individual learners. This variability in the existing literature makes it difficult to synthesize the evidence and identify the optimal way to implement retrieval practice in residency training. Most existing evidence also comes from single-center or small regional studies, and that further limits its external validity.

We aimed to address these literature gaps by designing a national prospective study testing a practical and scalable way of implementing retrieval practice: sending residents daily practice questions by automated text message. Evidence from small existing studies supports using text messaging to deliver practice questions [11,13-15], so we decided to test the technique on a larger and more generalizable scale.

This material was previously presented as a poster at the Association of Pediatric Program Directors annual meeting in Atlanta, GA, on March 27, 2025.

## Materials and methods

Study design

We designed a national prospective study to test how sending daily exam-style multiple-choice practice questions to pediatrics residents by text message would impact the residents’ performance on subsequent standardized exams. We adopted a quantitative, post-positivist orientation with a focus on an automated intervention and objective outcome measures to enhance generalizability. Through the Association of Pediatric Program Directors’ (APPD) Longitudinal Educational Assessment Research Network (LEARN), we recruited residency programs from across the United States to participate. Participating programs invited their first- and second-year residents to enroll in the study. Residents who chose to enroll provided their demographic information, cell phone number, and American Academy of Pediatrics (AAP) and American Board of Pediatrics (ABP) ID numbers to facilitate retrieving and matching their data.

We used a commercial bulk text messaging service (SimpleTexting, Chicago, IL) to automatically send daily text messages to participants. From August 2022 through June 2023, we sent a text message to each participating resident every Monday through Friday at 7:00 am in their home time zone. Each text message read, "Good morning! Click here to log in and answer your daily PREP question:" and contained a link to a specific question in the Pediatrics Review and Education Program (PREP), a bank of exam-style multiple-choice practice questions published by the AAP. The messages instructed the residents to click the link, log in to the PREP system, and answer that question. After residents logged in and selected their answers, the PREP system immediately provided feedback, including the correct answer and a detailed explanation of the question and the answer choices.

Data and metrics

The text messaging system logged the number - but not the identities - of individuals who clicked the link in each message. The PREP system logged the date and time each resident answered each question. We provided the AAP with a list of participating residents and a schedule detailing which specific question we sent to each individual resident each day of the study. The AAP used those data to determine when each participating resident answered each assigned question. That allowed us to count the number of study-assigned questions each participant answered within two days of receiving the text message.

After the daily text messages concluded, APPD LEARN provided each participant’s July 2022 and July 2023 scores on the ABP In-Training Exam (ITE): an annual standardized exam offered to all pediatrics residents, which has previously been shown to predict performance on the ABP board certification exam [16]. LEARN also provided July 2022 and July 2023 ITE scores for residents whose programs participated in the study but who individually chose not to enroll and from residency programs that did not participate in the study as control groups.

APPD LEARN staff de-identified all data before returning them to the authors for analysis. The overall study was reviewed and deemed exempt by the Biomedical Research Alliance of New York IRB under a reliance agreement from the Weill Cornell Medicine IRB (file # 22-12-057-380). IRB review and either approval or an exempt determination were also obtained at each institution that recruited residents and contributed data for the study.

Statistical analysis

We used descriptive statistics to characterize the study participants, with continuous variables summarized as mean (SD) or median (IQR) and categorical variables as frequency and percentage (n, %). We assessed associations between participants’ demographics and the number of questions they answered using the Wilcoxon rank sum or Kruskal-Wallis test, as appropriate, and we used univariate linear regression to examine the effects of demographic factors on continuous outcomes. We calculated Spearman rank correlations to assess associations between continuous variables. We performed all analyses in R software (version 4.2.3; R Development Core Team, Vienna, Austria), with two-sided p-values considered significant at an alpha level of 0.05.

## Results

Demographics

Thirteen residency programs, representing all regions of the United States and a range of program sizes, obtained IRB approval and joined the study. Table 1 lists the participating programs. From those programs, 293 residents enrolled in the study. Table 2 shows the demographics of all study participants. We initially intended for only first- and second-year residents to enroll in the study, but some third-year residents and one fourth-year resident also enrolled. Thus, we included them in analyses where we had sufficient data and excluded them from analyses that would have required data we did not have.

**Table 1:** Name, location, and size of the programs that participated in the study

Program	Location	Size (total number of residents)
Beaumont Health (Royal Oak)	Royal Oak, MI	24
East Tennessee State University	Johnson City, TN	24
Harlem Hospital Center	New York, NY	24
Medical University of South Carolina	Charleston, SC	52
Morehouse School of Medicine	Atlanta, GA	18
New York Presbyterian Hospital (Columbia Campus)	New York, NY	84
New York Presbyterian Hospital (Cornell Campus)	New York, NY	62
Oregon Health and Science University	Portland, OR	56
Our Lady of the Lake	Baton Rouge, LA	36
University of Illinois College of Medicine at Peoria	Peoria, IL	36
University of Texas Health Science Center at Houston	Houston, TX	72
University of Wisconsin Hospitals and Clinics	Madison, WI	48
Westchester Medical Center	Valhalla, NY	65

**Table 2:** Demographics of all residents who enrolled in the study

Category	Demographic	Number (Percent)
Postgraduate year in training	PGY-1	136 (46.4%)
	PGY-2	106 (36.2%)
	PGY-3	50 (17.2%)
	PGY-4	1 (0.3%)
Medical degree	MD	220 (75.1%)
	DO	70 (23.9%)
	MBBS	3 (1%)
Medical school	American	254 (86.7%)
	International	39 (13.3%)
Gender	Female	225 (76.8%)
	Male	68 (23.2%)
Age	<27	44 (15.6%)
	27-29	153 (54.3%)
	30-33	65 (23%)
	>33	20 (7.1%)

Text messaging and response rates

**Figure 1:**
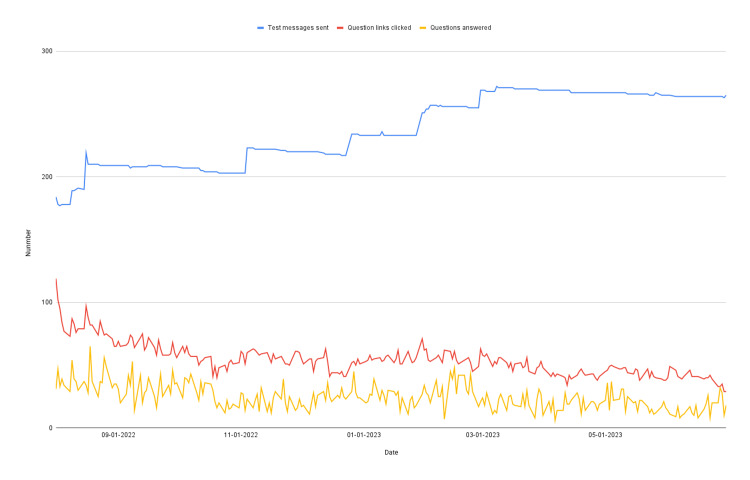
Number of text messages sent, number of links clicked in the text messages, and number of study-assigned PREP (review) questions answered by participants each day over the course of the study PREP: Pediatrics Review and Education Program

Over the course of the study, we sent out an average of 237 text messages to participating residents per day (IQR: 209-266). That is less than the total of 293 residents who enrolled in the study because some residents joined after the study was underway and some residents withdrew mid-study. Residents enrolled in the study clicked the link to the practice question in an average of 54 text messages per day (IQR: 45-60), and they answered an average of 24.8 study-assigned PREP questions per day (IQR: 17-30). Figure 1 shows how the daily number of text messages sent, links clicked, and questions answered varied over the course of the study.

**Figure 2:**
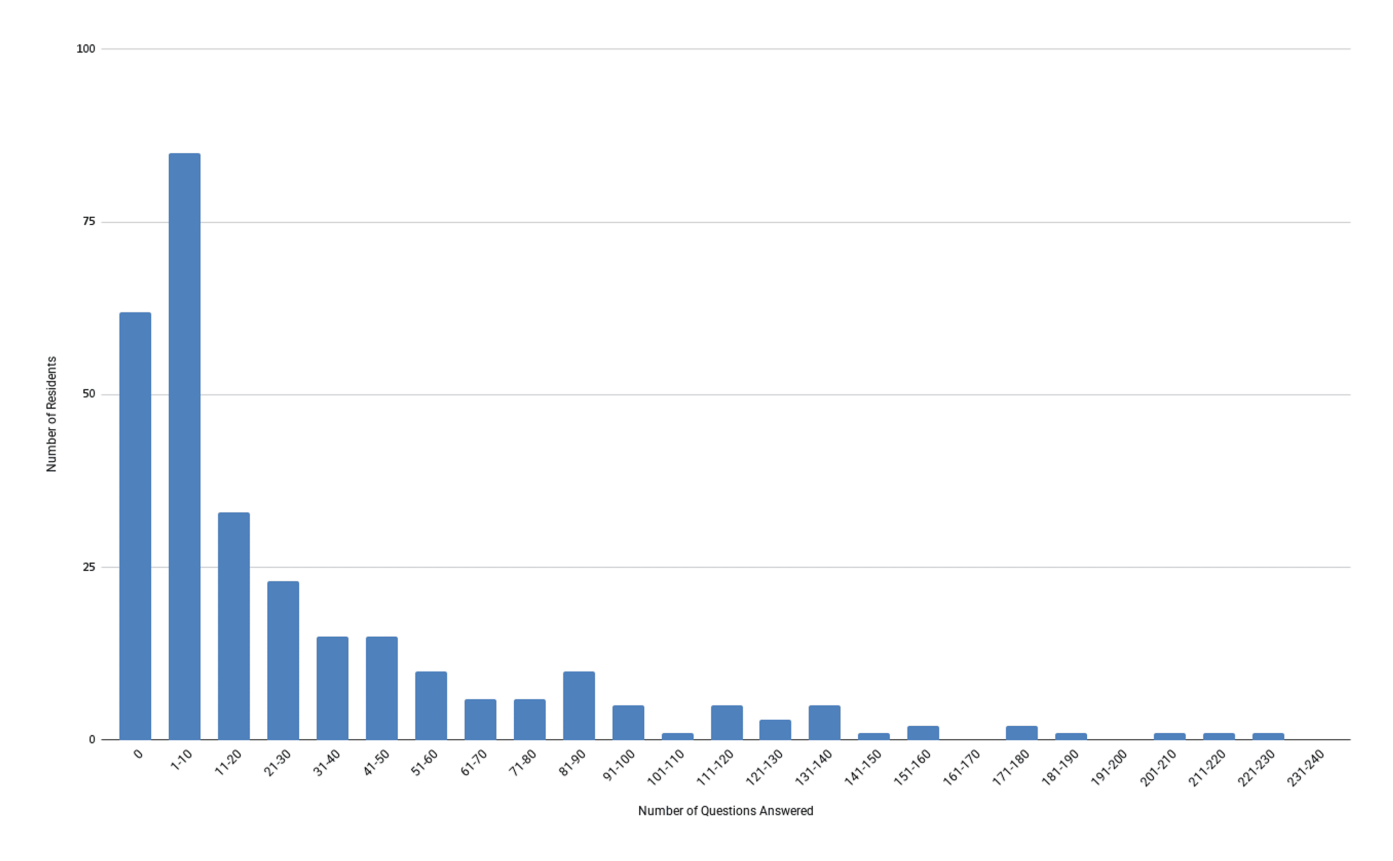
Total number of study-related PREP questions answered by residents during the study PREP: Pediatrics Review and Education Program

Over the course of the study, individual residents answered an average of 34 of their assigned questions within two days of receiving the text message (IQR: 27.8-40). Figure 2 shows the breakdown of how many residents answered how many total questions. Of note, 21% of participants (62) never answered a single question, and 50% of participants (147) answered 20 questions or fewer.

We assessed whether any of the demographic factors we collected correlated with the number of PREP questions that participants answered. There was no significant association between the number of questions residents answered and their PGY level, gender, age, or American vs international medical degree; p > 0.2 in all cases. There was no significant correlation between residents’ performance on the 2022 ITE and the number of questions they answered during the study (p = 0.07). Two factors did correlate with residents’ engagement with the practice questions: residents with a DO degree answered significantly more questions than residents with an MD (average 41.6 vs 25.3, respectively; p = 0.043), and residents at medium-sized programs (31-60 total residents) answered more questions than residents at small (≤ 30 residents) or large (> 60 residents) programs (51.0 vs 21.2 and 20.0, respectively; p = 0.019, and p=0.002).

Effects of practice questions

In designing this study, we assumed that - based on results of prior studies - the daily text messages would induce residents to answer more PREP questions, and we intended to analyze how answering more PREP questions might impact the residents’ ITE scores. Unfortunately, so few residents answered so few questions that analyzing our initial research question is likely futile. Nevertheless, we assessed the overall effectiveness of the text messaging intervention by comparing 2022 and 2023 ITE scores between residents who enrolled in the study and a control group of all other US pediatrics residents whose programs did not participate in the study. In 2022, before the start of the study, there was no significant difference between participating residents’ and non-participating residents’ median ITE scores: 151 and 149, respectively; p = 0.8 by the Wilcoxon rank sum test. In 2023, at the conclusion of the study, residents who had participated in the study scored statistically significantly, but only very slightly, lower than controls on the ITE: 156 vs 159, respectively (p = 0.038). Using each resident as their own control by comparing the changes in their ITE scores from 2022 to 2023, however, there is no significant difference; participants' and non-participants' ITE scores both increased by a median difference of 9 and 10 points, respectively (p = 0.7).

Despite having so few participants answer so few questions, we attempted to test the relationship between PREP questions and ITE scores using bivariate analyses; neither the number of PREP questions residents answered nor the percentage of those questions they answered correctly significantly correlated with the change in their ITE scores (p = 0.8 for both). Even restricting the analysis to the 61 residents who engaged meaningfully with the intervention by answering ≥50 questions, we found no significant difference in 2022 to 2023 ITE score changes between participants and controls (p > 0.9). Analyzing just those 61 residents, there was still no significant correlation between PREP questions answered and ITE score change (p = 0.25).

## Discussion

Small and retrospective studies have shown that retrieval practice, particularly using exam-style multiple-choice practice questions, can be a powerful learning strategy for medical students and residents [3,4,8,11,12]. Other studies have shown that text messaging can be an effective tool for delivering retrieval practice questions [13-15]. The variability and limitations of the existing literature make it challenging to synthesize the evidence and identify the optimal way to implement retrieval practice in graduate medical education, though, so we attempted to test this approach in a national prospective study. We used automated daily mass text messages to direct a national sample of pediatrics residents to answer targeted PREP questions, and we intended to test how answering more practice questions would impact their annual standardized exam scores.

We found that the text messages did not induce residents to answer more PREP questions, and we were therefore unable to fairly test our primary research question. Several previous studies have found that delivering PREP questions via text message was effective, but they were small studies in contexts where the learners receiving the text messages likely had personal relationships with the educators sending them [14,15]. The importance of those relationships for the effectiveness of text messaging as an educational tool may have previously been underappreciated. In our study, we sent generic mass text messages to a national group of residents who had no relationship with the researchers; the impersonal nature of our approach may have contributed to its ineffectiveness.

Our unsuccessful intervention - the fact that residents did not answer PREP questions sent to them via text message - is one major limitation of this study. Sample size is another limitation; while 293 participants are a large group compared to most other prospective educational studies, it still represents only a small fraction of the total population of pediatric residents in the United States.

Further research is needed to identify effective ways of encouraging residents to answer more exam-style practice questions. As an intermediate step, qualitative studies exploring residents’ perceptions and motivations around retrieval practice with exam-style practice questions could provide a foundation for identifying and testing strategies to increase residents’ engagement. After we find a reliable way to increase residents’ engagement with retrieval practice, we will then be able to fairly test how retrieval practice impacts exam performance on a large and generalizable scale.

## Conclusions

Residents have limited time and much to learn. Small retrospective studies have found that retrieval practice using exam-style multiple-choice practice questions can be a powerful aid to that learning and that text message reminders can effectively boost residents’ engagement with retrieval practice. We aimed to test that approach in a national prospective study, but our intervention was unsuccessful. We sent automated daily mass text messages to residents directing them to answer practice questions, but few participants answered few questions, so we were unable to test how answering more practice questions might impact residents’ medical knowledge. Future research needs to explore motivators and barriers to residents’ engagement with retrieval practice in order to identify effective strategies. Only then will we be able to rigorously test how retrieval practice impacts medical knowledge and skills.
